# Spontaneous Resolution of a Full-Thickness Macular Hole: A Complex History

**DOI:** 10.7759/cureus.82999

**Published:** 2025-04-25

**Authors:** Christopher Stewart, Jaskaran S Bhangu, Mahmoud Awad, Gwyn Williams

**Affiliations:** 1 Ophthalmology, Swansea University, Swansea, GBR; 2 Ophthalmology, Singleton Hospital, Swansea, GBR

**Keywords:** full-thickness macular hole, proliferative diabetic retinopathy (pdr), retinal detachment surgery, secondary macular hole, traumatic macular hole

## Abstract

Macular holes are vision-threatening retinal conditions that can significantly affect a patient's quality of life. Secondary macular holes, often associated with intricate ocular histories, create significant obstacles in effective patient management. This case report discusses a gentleman in his 60s, whose routine monitoring for proliferative diabetic retinopathy revealed the emergence of a small full-thickness macular hole, likely worsened by his complex ocular history. His ocular history included a pars plana vitrectomy combined with heavy silicone oil tamponade following a traumatic retinal detachment three years earlier, alongside pan-retinal photocoagulation, anti-vascular endothelial growth factor and steroid injections for his diabetic eye disease. Spontaneous closure of the full-thickness macular hole within three months not only improved visual acuity from 1.04 LogMAR to 0.68 LogMAR but also illustrates the retina's natural reparative capabilities. This underscores the complex aetiology of secondary full-thickness macular holes, highlighting the interplay of trauma, proliferative diabetic retinopathy, anti-vascular endothelial growth factor treatments, and prior surgeries. Observation, especially in smaller secondary holes, can be a practical approach, using the retina's natural reparative processes while avoiding surgical risks.

## Introduction

Macular holes represent critical disruptions in the neuro-retinal architecture, predominantly caused by idiopathic factors and associated with vitreomacular traction during posterior vitreous detachment [1]. The incidence of a macular hole in a predominantly Caucasian population lies at around 7.8 per 100,000 per year [2]. The predominant risk factors for developing an idiopathic macular hole include advancing age, female gender, and cataract surgery whilst obesity has been shown to be protective against the disease [3,4]. Our current understanding for older age leading to macular hole is that over time there is vitreous degeneration (synchysis) concurrently with vitreoretinal adhesion weakening (syneresis) that in combination can result in a spectrum of vitreomacular disorders which include macular hole [5]. The link with females is attributed to the hormonal changes with oestrogen [6]. In a study by Wergenthaler et al., the presence of oestrogen is protective to the neuroretinal cell layers, and the sudden drop seen in menopause could be the underlying reason behind the greater risk in women [6].

The traditional classification of macular holes was based on clinical observations seen during fundoscopic examinations using Gass staging [7]. Recent advancements in imaging technology, particularly optical coherence tomography, have revolutionized the classification of macular holes, providing greater specificity in diagnosis and tailoring of treatment approaches. The International Vitreomacular Traction Study categorised holes based on the minimum linear diameter; this was small (< 250 μm), medium (≥ 250-400 μm), and large (> 400 μm) [7]. At present, medium holes or smaller have nearly 100% closure rates with corresponding improvement in best corrected visual acuity through surgical intervention [7]. This classification was taken one step further by the CLOSE study group who subdivided larger holes into large > 400 to ≤ 550 µm, X-large >550 to ≤ 800 µm, XX-large >800 to ≤ 1000 µm, and giant >1000 µm [7]. The variability in outcomes seen in larger holes is significant; however, innovative new surgical techniques have expanded the possible interventions for these cases, providing better management options. These new techniques have encompassed macular hydro-dissection, human amniotic membrane graft, and autologous retinal transplantation [7]. The underlying focus across all the different classification systems has been the correlation between size of the macular hole and surgical outcomes thus aiding in prognosis and treatment planning. Non-surgical techniques, such as laser and pharmacological treatments like ocriplasmin, have demonstrated efficacy in select cases, along with active observation for spontaneous closure [8].

Macular holes can also be classed based on the underlying pathophysiology. Idiopathic are due to the vitreomacular traction which are closely related to another type called myopic macular holes. The other types are secondary macular holes and traumatic macular holes that are distinguished from one another in the literature [7]. Secondary macular holes all have distinct features and pathogenesis and are discussed individually. The following are known secondary macular hole causes: macular telangiectasia, retinal artery macro aneurysm, cystoid macular oedema, ocular surgery, and finally laser devices used to treat other ocular disease [7]. The literature and evidence for all forms of secondary macular holes are outside the scope of this case report. It is important to be aware that each type of secondary macular hole has separate mechanisms and thus can differ in their response to treatment. The final classification comprises traumatic macular holes, which are relevant to the case being discussed.

## Case presentation

A male patient in his 60s with a known proliferative diabetic retinopathy (PDR) presented for a routine follow-up assessment of diabetic eye disease. He reported a six-week history of reduced vision, metamorphopsia, and difficulty recognising faces, especially when covering his right eye. Best corrected visual acuity (BCVA) was recorded as 1.04 LogMAR in the left eye and 0.34 LogMAR in the right. Fundoscopic examination demonstrated bilateral R3AM1A grading, indicative of proliferative diabetic retinopathy with maculopathy.

His past medical history included type 2 diabetes mellitus, diagnosed five years earlier, and mixed dyslipidaemia. Glycaemic control was suboptimal, with a recent hemoglobin A1C (HbA1C) of 54 mmol/mol (20-42). His diabetes was managed with a combination of daily insulin therapy, empagliflozin 25 mg once daily, and metformin 1 g twice daily.

**Figure 1:**
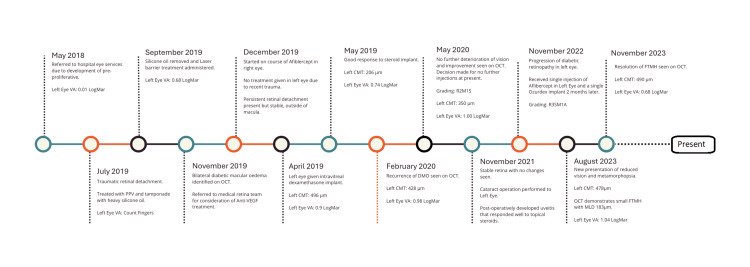
Timeline of patients ocular history including central macular thickness (CMT) and best corrected visual acuity (BCVA) alongside clinical interventions. OCT: optical coherence tomography, PPV: pars plana vitrectomy, FTMH: full-thickness macular hole, DMO: diabetic macular oedema, MLD: minimum linear diameter

The patient had a complex ocular history, which is visually summarised by a timeline in Figure 1. He was first diagnosed with pre-proliferative diabetic retinopathy four years prior to his current presentation. One year later after this diagnosis, he sustained a traumatic retinal detachment in the left eye secondary due to blunt orbital trauma caused by being hit in the eye. Surgical intervention included pars plana vitrectomy, cryotherapy, and tamponade with heavy silicone oil (Densiron), which was removed after 10 weeks. The injury resulted in subretinal and vitreous haemorrhages, with a deterioration in BCVA from 0.01 to 0.68 LogMAR. Subsequent fundus examination revealed a persistent but stable shallow supero-temporal retinal detachment, not involving the macula. Laser barrier treatment was administered to prevent progression into the macular region which was successful. The patient was then seen to have developed bilateral diabetic macular oedema (DMO). The right eye was managed with three intravitreal injections of aflibercept (Eylea, 2 mg). In the left eye, DMO was treated with an intravitreal dexamethasone implant (Ozurdex). The implant was placed in the inferonasal vitreous cavity to minimise interaction with the residual supero-temporal detachment. The treatment response was favourable, with a reduction in central macular thickness (CMT) from 496 µm to 206 µm and an improvement in BCVA from 0.90 to 0.74 LogMAR within four weeks. Intraocular pressure (IOP) remained stable, and visual field testing revealed a superior hemifield defect and inferior scotomas consistent with retinal scarring. Six months later, recurrence of macular oedema was observed in the left eye, with CMT increasing to 428 µm and BCVA declining to 0.98 LogMAR. Given the absence of significant visual deterioration, a conservative approach with observation was adopted. Over the following months, BCVA remained stable at 1.00 LogMAR, with a marginal reduction in CMT to 350 µm. The patient subsequently underwent an uneventful cataract extraction in the left eye. Post-operatively he did develop uveitis and this was managed effectively with a short course of topical corticosteroids. One year following cataract surgery, the patient demonstrated progression of diabetic retinopathy in the left eye, from R2M1S to R3SM1A, characterised by active neovascularisation and worsening macular oedema. Management included a single intravitreal aflibercept injection and pan-retinal photocoagulation. A second Ozurdex implant was then administered two months after a poor response to aflibercept.

**Figure 2:**
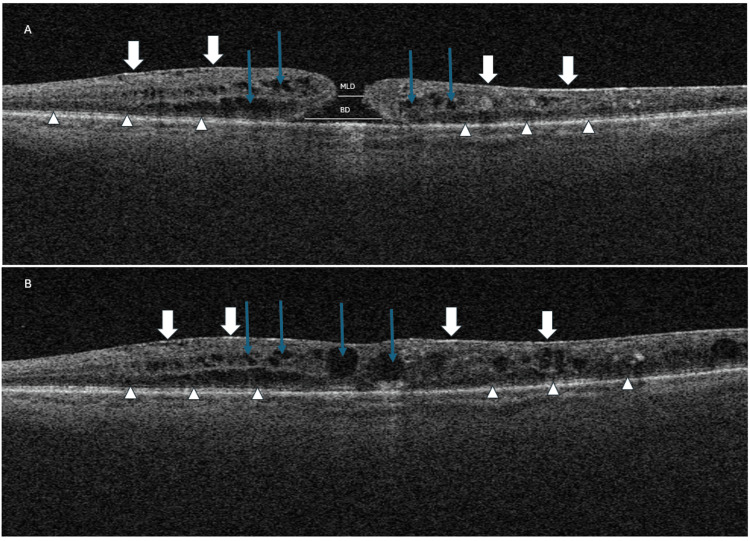
Optical coherence tomography showing (A) initial full thickness macular hole with a minimum linear diameter (MLD) of 183 µm and base diameter of 804µm. (B) shows the resolution of the macular hole. The Ellipsoid Zone (EZ) is highlighted with the white arrow heads demonstrating a disruption and absence near the macular hole. The white arrows demonstrate the presence of an epiretinal membrane seen on both scans. The blue arrows indicate intraretinal cystoid changes that are present.

Returning to the current presentation of reduced vision, a full ocular assessment was conducted. Fundoscopy and optical coherence tomography demonstrated the development of a full-thickness macular hole, measuring 178 microns in minimum linear diameter, as shown in Figure 2A. Adhering to the CLOSE study parameters, this corresponds to a small macular hole [7]. The success rates for surgical intervention for a hole of this size exceed 90% [9]. The standard approach is pars plana vitrectomy and intravitreal gas tamponade with an internal limiting membrane peel.

Once the macular hole was identified, the decision was made to undergo a period of observation (two to three months) before considering surgery. Spontaneous closure rates vary significantly, between 2.5% and 27% [9]. Compared to surgical intervention, observation may result in worse outcomes, especially as delaying surgery beyond the duration the hole has been present has been associated with vision loss [9]. Features in the history that supported surgery were the short duration of symptoms, the pseudophakic status of the eye, disruption to the ellipsoid zone (EZ), and notably the small minimum linear diameter (MLD). The drawbacks of immediate intervention centred around the active DMO in an eye that had a pre-existing retinal detachment, and the presence of an epiretinal membrane (ERM). Although surgery was offered, the patient opted for conservative management with active observation. At the time, he had a maximum retinal thickness of 478 microns with diffuse, centrally involving intraretinal fluid. The hope with observation was that the macular oedema would settle, and the macular hole would spontaneously close. 

After three months, spontaneous full-thickness closure occurred, as demonstrated in Figure 2B, improving visual acuity to 0.68 LogMAR from 1.04. The patient also reported a noticeable absence of metamorphopsia, with no distortion noted on the Amsler grid. Quantification of the distortion was not performed using M-charts but was subjectively significant. This reflects findings by Takeyama et al., who demonstrated that macular hole diameter is a key predictive factor for metamorphopsia following hole resolution [10]. The decision to forgo surgical intervention avoided associated risks, highlighting the potential for non-invasive management in selected cases.

## Discussion

Macular holes, particularly secondary ones, arise from complex mechanical, biochemical, and cellular dynamics at the vitreomacular interface [7]. In this case, the delayed onset following trauma suggests progressive vitreoretinal changes, including vitreoschisis, tangential traction, and post-vitrectomy remodelling, as described by Lipham et al. [11]. The presence of active PDR adds complexity through fibrovascular proliferation and the generation of biochemical mediators such as vascular endothelial growth factor (VEGF). VEGF not only influences retinal neovascularisation but also modulates extracellular matrix remodelling, potentially precipitating or resolving tractional forces at the macula [12,13]. Anti-VEGF treatments, integral to managing PDR, may further alter the microenvironment, potentially facilitating spontaneous closure by stabilising fibrovascular activity and relieving tractional stress. For example, restoration to the EZ through reconstruction of the external limiting membranes has been theorised by Chaturvedi et al. [14]. Studies have shown that diabetic patients have a greater incidence of vitreomacular disorders such as epiretinal membranes and vitreomacular adhesion compared to non-diabetic patients [13]. This suggests that the diabetic microenvironment influences the structural integrity of the macula and its susceptibility to hole formation. This is seen in our case, by the presence of the ERM alongside an absent EZ.

The spontaneous closure rate of secondary macular holes, particularly those associated with trauma, has been reported to be higher than that of idiopathic macular holes [15]. It is well established that blunt ocular trauma can cause macular holes, defined as traumatic macular holes. Although the exact mechanism remains uncertain, several theories exist. The first two relate to acute tractional and shape changes the globe undergoes, causing stress at the vitreoretinal interface [16]. Another theory postulates that retinal atrophy occurs over time, though this would suggest a more delayed mechanism [16]. While macular hole formation after trauma typically occurs immediately, there is evidence of a delayed onset of up to several weeks but none that extend into years like in this instance [16].

The impact of prior vitrectomy on macular hole development and closure is an important consideration in this case. Okonkwo et al. found that macular hole formation following vitrectomy for retinal detachment occurred in 1.1% of cases, with a mean time to diagnosis of one week to three months post-surgery [17]. This highlights the potential long-term effects of retinal reattachment surgery on macular structure and function.

OCT biomarkers play a crucial role in predicting spontaneous closure of macular holes. Kusuhara et al. identified several OCT parameters associated with spontaneous closure, including smaller minimum linear diameter, absence of a full-thickness defect, and presence of an ERM [18]. Another feature that was seen on both the initial and follow-up OCT for our patient was a disruption to the EZ, shown in Figure 2. The integrity of the EZ, marking the interface between the inner and outer segment of photoreceptors, has been established as important not only in macular hole outcomes but also in other retinal conditions such as diabetic macular oedema. Studies have identified that patients with an intact EZ have significantly better visual acuity [19].

These findings on OCT can guide clinical decision-making and help identify patients who may benefit from observation rather than immediate surgical intervention. This scenario aligns with existing literature, indicating that smaller secondary macular holes with limited traction and short durations are more likely to close spontaneously [7,15]. Observational strategies in such cases avoid surgical risks, including retinal detachment, recurrence, iatrogenic trauma, or cataract formation. However, the decision to observe should be individualised, balancing the benefits of potential spontaneous resolution against the risk of progressive visual deterioration. Evidence suggests that while surgical closure rates are higher, visual acuity improvements between surgical and observation groups can be comparable [20]. This supports the consideration of observation in carefully selected patients with secondary macular holes and highlights that successful closure does not always correlate with improved best-corrected visual acuity.

## Conclusions

Our case, although presenting no novel findings, encapsulates a variety of risk factors for hole formation. It serves as an excellent educational review of macular holes, their causes, and pathophysiology. The spontaneous closure demonstrates that the size of the full-thickness macular hole remains the greatest indicator for successful outcomes for closure, irrespective of the cause. Secondary macular holes, particularly those linked to trauma or PDR, highlight the intricate interplay of ocular pathologies. Surgical intervention remains the gold standard treatment for macular holes, however, an emphasis for the potential of observation in patients with complex pre-existing retinal pathology should be considered in select cases.
